# One-Dimensional ^13^C NMR Is a Simple and Highly Quantitative Method for Enantiodiscrimination

**DOI:** 10.3390/molecules23071785

**Published:** 2018-07-20

**Authors:** Peter P. Lankhorst, Jozef H. J. van Rijn, Alexander L. L. Duchateau

**Affiliations:** DSM Biotechnology Center, P.O. Box 1, 2600 MA Delft, The Netherlands; joep.rijn-van@dsm.com (J.H.J.v.R.); lucien.duchateau@dsm.com (A.L.L.D.)

**Keywords:** enantiomers, Pirkle’s alcohol, TFAE, qNMR, mandelonitrile, CSA, chirality, chiral solvating agent

## Abstract

The discrimination of enantiomers of mandelonitrile by means of 1D ^13^C NMR and with the aid of the chiral solvating agent (S)-(+)-1-(9-anthryl)-2,2,2-trifluoroethanol (TFAE) is presented. ^1^H NMR fails for this specific compound because proton signals either overlap with the signals of the chiral solvating agent or do not show separation between the (S)-enantiomer and the (R)-enantiomer. The ^13^C NMR method is validated by preparing artificial mixtures of the (R)-enantiomer and the racemate, and it is shown that with only 4 mg of mandelonitrile a detection limit of the minor enantiomer of 0.5% is obtained, corresponding to an enantiomeric excess value of 99%. Furthermore, the method shows high linearity, and has a small relative standard deviation of only 0.3% for the minor enantiomer when the relative abundance of this enantiomer is 20%. Therefore, the ^13^C NMR method is highly suitable for quantitative enantiodiscrimination. It is discussed that ^13^C NMR is preferred over ^1^H NMR in many situations, not only in molecules with more than one chiral center, resulting in complex mixtures of many stereoisomers, but also in the case of molecules with overlapping multiplets in the ^1^H NMR spectrum, and in the case of molecules with many quaternary carbon atoms, and therefore less abundant protons.

## 1. Introduction

The enantiomers of chiral compounds often behave differently in biological systems, for instance pharmaceutical products in the human body. For that reason, it is important in organic synthesis to avoid racemic mixtures and to synthesize optically pure compounds. As a consequence, analytical methods are also needed that can discriminate between the two enantiomers of a chiral compound, or more stereoisomers if more than one chiral carbon atom is present in the molecule. NMR is a very powerful technique for discrimination of diastereomers and enantiomers [1]. In the case of enantiomers, the NMR spectrum is not different for the two enantiomers because the most frequently used solvents do not provide a chiral environment. However, various methods exist to create a chiral environment resulting in different NMR spectra for the two enantiomers. A comprehensive review on these methods can be found in Ref. [1], and a recent review on the use of chiral solvating agents for NMR can be found in Ref. [2].

Reaction with a chiral derivatization agent (CDA) is one method, addition of a chiral solvating agent (CSA) is another method. (S)-(+)-1-(9-anthryl)-2,2,2-trifluoroethanol (TFAE), also known as Pirkle’s alcohol [3], is the most frequently used CSA, and, in our view, the most versatile one. The very acidic CHOH proton and the hydroxyl proton of TFAE are able to form hydrogen bonds with functional groups of the compound of interest and the multiple aromatic rings can strengthen the complex through hydrophobic interaction [4]. At the same time, the aromatic rings introduce shielding or deshielding on protons of the compound of interest, and the geometry of the association complex, as well as the equilibrium constant of the formation of the complex determined how much a particular proton is shielded or deshielded. This amount of shielding or deshielding will be different for the S-enantiomer and the R-enantiomer of the compound of interest. Many publications have appeared demonstrating the successful application of TFAE as CSA in combination with ^1^H NMR (see, for instance, Refs. [5,6,7], and references given in [8]). It should be noted here that one limitation of the method is that the hydrogen bonding of CSA with a target molecule is only strong enough in very apolar solvents, and therefore the method is restricted to compounds that are soluble in chloroform, or similar apolar solvents [9].

^13^C NMR has only recently been used for this purpose [10]. ^13^C NMR has long been ignored for enantiodiscrimination, probably because of the low sensitivity of ^13^C NMR, although it has been used for discrimination of diastereomers [11,12]. With the advent of high field NMR spectrometers and sensitive probes with cryogenically cooled ^13^C coils, there is no reason to avoid ^13^C NMR any longer. Very recently, we have shown that one important advantage of 1D ^13^C NMR is the extremely high resolution that can be obtained, which led to the discrimination of all eight stereoisomers (four diastereomeric pairs of enantiomers) of the three-chiral-carbon containing α-tocopherol (Vitamin E). We could assign all eight stereoisomers, and 0.75% of a minor stereoisomer could be detected in a sample of pure RRR α-tocopherol in an NMR experiment of 30 min (256 scans) [13]. The use of multinuclear NMR for enantiodiscrimination has been reviewed recently [14].

In this contribution, we want to show that ^13^C NMR is not only a preferred method in the case of complex mixtures of many stereoisomers with overlapping multiplet signals in the ^1^H spectrum, but also in the (at first glance) relatively simple case of (R,S)-mandelonitrile, for the simple reason that ^13^C NMR offers more resolution, since broadband decoupling removes overlapping multiplets, and that quaternary carbon atoms also can contribute to increasing the chance to find a suitable probe in the spectrum for detection of the two enantiomers. Alternative approaches to the problem of overlapping multiplets in the ^1^H spectrum are the use of homonuclear decoupled (pure shift) ^1^H spectra [15], or pure shift Heteronuclear Single Quantum Coherence (HSQC) spectra [16]; however, these methods do not detect quaternary carbons and are not implemented easily for routine use.

Various analytical methods are reported for the chiral analysis of mandelonitrile. A variety of derivatized cyclodextrins was used for the determination of the optical purity of mandelonitrile by ^1^H NMR in aqueous solution [17]. The best separation was achieved with acetyl-β-cyclodextrin (*K*_d_ = 12 dm^3^ mol^−1^). A straightforward integration was possible if there was more than 5% of one of the enantiomers in the solution. Below this value, the results were not reproducible enough for quantitative analysis because there was no baseline separation of the two signals. Chiral chromatography is currently most often used for chiral analysis of mandelonitrile. Several high-performance liquid chromatography (HPLC) methods have been described in which different types of chiral stationary phases (CSPs) are used: Chiralcel OB-H [18], Chiralcel OJ-H [19,20,21,22], Sumichiral OA-4400 [23], Chiralpak IA [24] and Chiralcel OD-H [25]. These stationary phases were used in combination with either mixtures of n-hexane and isopropanol [18,19,20,21,22,23,24,25], n-hexane, ethanol and 1,2-dichloroethane [23], or n-hexane, isopropanol and trifluoroacetic acid [24]. By means of these chiral HPLC methods, enantiomeric excess values of mandelonitrile up to 99% could be established [18,20].

Enantiomeric excess determination of mandelonitrile by means of chiral gas chromatography has also been reported. For the separation of mandelonitrile enantiomers, different types of derivatives are made which are analyzed on a range of capillary gas chromatography (GC) columns: trifluoroacetyl derivatives on Chiraldex G-PN (gamma-cyclodextrin, propionyl) [26], trimethylsilyl derivatives on Chiraldex G-TA (gamma-cyclodextrin, trifluoroacetyl) [27], propionyl derivatives on Supelco Beta Dex 325 [28] and acetyl derivatives on Chirasil-Dex CB [29].

## 2. Results

**Figure 1:**
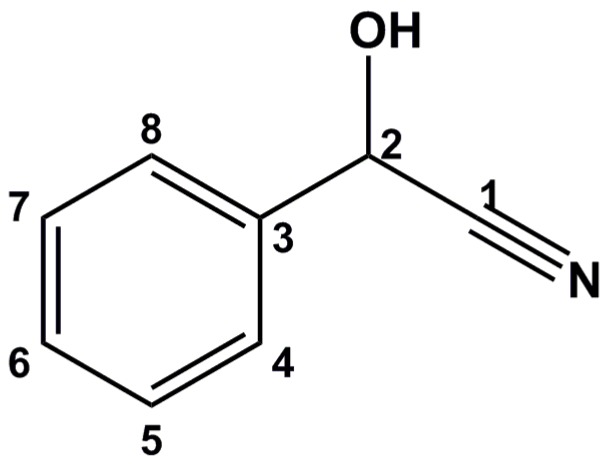
Structure of mandelonitrile.

The structure of mandelonitrile is shown in Figure 1. Our attempts to obtain resolved signals for the racemate by means of ^1^H NMR were not satisfactory because the aromatic protons overlap with the proton signals of TFAE, and the CH(OH) proton does not show a splitting of signals. The signal of the hydroxyl proton shows two partially resolved, but broadened signals at 240 K. The resolution of these broadened signals was insufficient for accurate enantiomeric excess determination.

**Figure 2:**
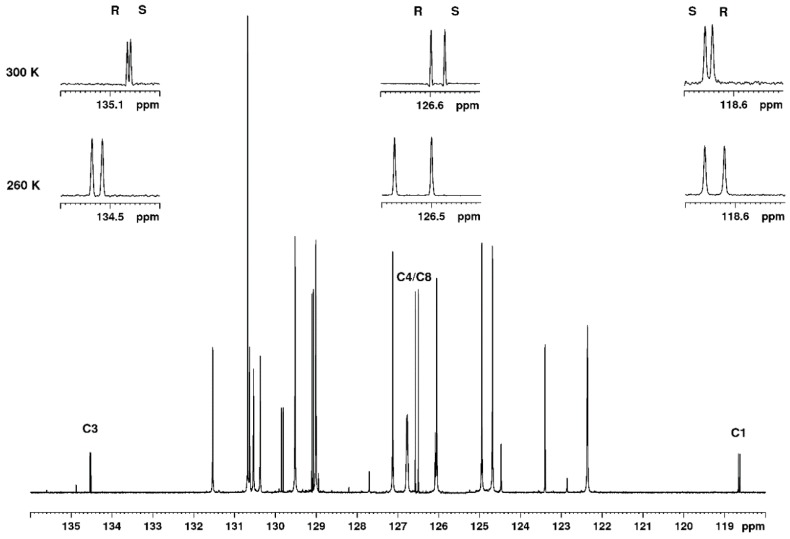
Low field region of the ^13^C NMR spectrum of a mix of R and S mandelonitrile, 260 K with TFAE. The expansions show the signals of from left to right C3, C4/C8 and C1 at 260 K and at 300 K. The assignment of R and S is given.

The particular advantage of ^13^C NMR in this and similar cases is that, as a result of broadband decoupling, all signals appear as singlets, and, moreover, that quaternary carbon atoms may be used as well for the analysis. The ^13^C NMR spectrum at two different temperatures is shown in Figure 2, and it is clear that, even at 300 K several carbon signals, including the quaternary nitrile carbon at 118.6 ppm and the quaternary aromatic carbon at 134.5 ppm, appear as a resolved pair of signals representing the two enantiomers. The signal of C4/C8 has a higher intensity because of the symmetry of the phenyl ring and has no other signals in close vicinity. Therefore, this signal was chosen for the analysis, and, in the forthcoming paragraphs, we will show the extremely linear response of the ratio of the two signals to the R/S ratio. Furthermore, the limit of detection (LOD) and the repeatability of the method will be addressed. It is clear from Figure 2 that the separation of signals is larger at the lower temperature. At temperatures below 260 K, some line-broadening was observed, and, therefore, 260 K was chosen as the optimal temperature for the quantitative enantiodiscrimination.

It should be noted that, in general, one can fully trust that the ratio of signals given by NMR, whether ^1^H or ^13^C NMR, of the two enantiomers are quantitative, even if the NMR spectra are recorded with a short delay. It has been suggested recently that ^13^C NMR is less useful for the quantitative analysis of the enantiomeric ratio than ^1^H NMR [30]; however, this restriction is not valid because the relaxation times and nOe’s of like carbon atoms are the same for the R and the S enantiomer. For the case of diastereomers, this has been shown recently by Otte et al. [31]. Here, we show convincingly that indeed the ^13^C NMR method is very reliable for this purpose by presenting the validation parameters of the method.

### 2.1. Linearity and Quantitative Trueness of Enantiodiscrimination by 1D ^13^C NMR

Before preparing artificial mixtures of the racemate (R/S) and R mandelonitrile, it was important to know the absolute chemical purity of both reference samples. If the two reference samples have different purities, the true ratio in the mixture will be different from the ratio calculated from the weights alone. The best method to obtain the purity of the samples is by means of quantitative NMR (qNMR) [32,33]. The qNMR method has been described and applied many times. Indeed, we found that the racemate has a purity of 88.8% and the pure R compound has a purity of 81.4%. Furthermore, one cannot trust that the pure R compound is really 100% pure, and that the racemate is exactly racemic. Therefore, we tested the R mandelonitrile by means of the ^13^C NMR method and found that it contains 2.4% of the S enantiomer, while we found that the racemate is truly racemic within the limits of the method. The R/S ratio is 49.9/50.1. These observations imply that, when preparing artificial mixtures by weight, one has to correct the weights of the two ingredients of the mixture for their R/S ratio and for their chemical purity in order to obtain the true theoretical (= predicted) value of the % S in the mixture. It is a particular advantage of using NMR for enantiodiscrimination that the absolute chemical purity can be obtained with the same technique without the use of a reference sample of that compound.

**Figure 3:**
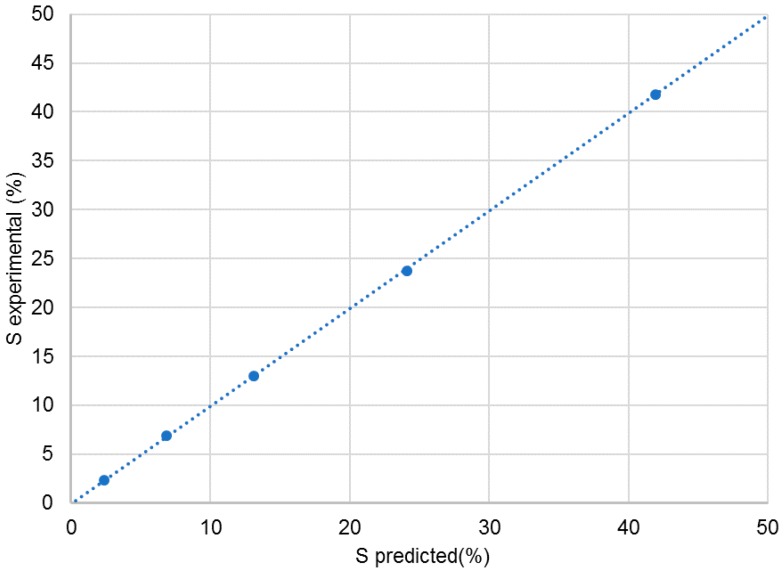
Percentage of S enantiomer by experiment versus predicted percentage.

Next, the mixtures were dissolved in CDCl_3_, and NMR spectra were recorded as described in the Materials and Methods section. The experimental % S was obtained by integration of the two signals at 126.5 ppm after careful phasing and baseline correction. Table 1 shows in column A the theoretical % S obtained from the weight of the two compounds in the mixture. In column B, the corrected % S is shown, after taking into account the enantiomeric purity and the chemical purity of the two compounds, and, in the last column, the % S is shown that was found experimentally. A graph of the correlation between experimental and theoretical value is shown in Figure 3. The relation is extremely linear with a correlation coefficient of 0.9999, an intercept close to 0 and a slope of 1. One further remark on the quantitative trueness of this ^13^C NMR method for the purpose of enantiodiscrimination must be made: the R/S ratio of the racemate was determined, as 49.9/50.1, which is the expected ratio, showing already convincingly, that short interpulse delays may be used in this case. The ultimate evidence for the quantitative trueness of the method was obtained by determining the T1 values of the carbons of the R and S enantiomer. All corresponding carbons have the same T1 value, those of C4/C8 are: 2.1 s.

### 2.2. Limit of Detection (LOD)

**Figure 4:**
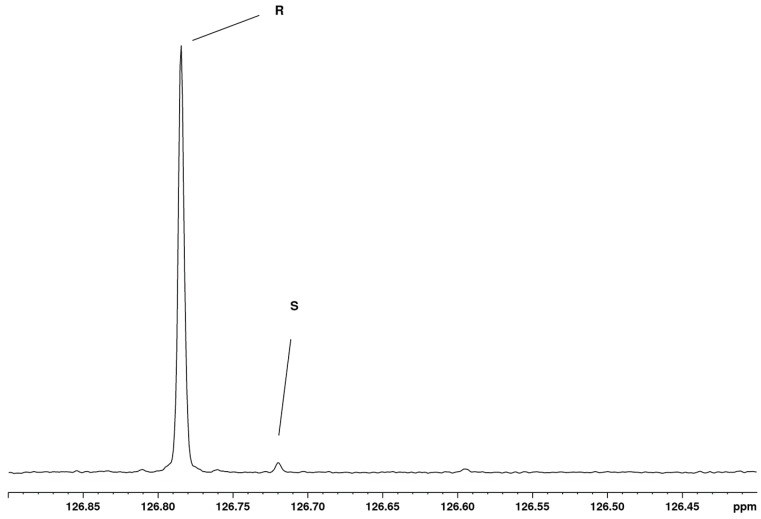
Expansion of 0.5 ppm of the ^13^C NMR spectrum of the “pure” R enantiomer of mandelonitrile. The region of interest, where the signals of C4/C8 appear, is shown.

The limit of detection (LOD) was estimated from the sample containing “pure” R enantiomer. The relevant part of the ^13^C NMR spectrum of this sample is shown in Figure 4, and it is evident that the “pure” R sample contains 2.4% of the S enantiomer, and the signal-to-noise ratio of the S peak is 16. By extrapolation, one can conclude that 0.5% of the S enantiomer would give an S/N ratio of 3, which is generally considered as the LOD [34].

At the same time, Figure 4 is a demonstration of the high resolution obtained in the 1D ^13^C NMR spectrum, as the peak separation is only 0.065 ppm (11 Hz), a very small separation when compared to the full range of 200 ppm of the ^13^C NMR spectrum. However, this separation is more than sufficient for the purpose of discrimination and quantitation, and it is clear that also at lower field strengths the separation of the two signals would be sufficient to enable their discrimination and quantitation. Of course, the sensitivity will be less at lower field strengths, leading to longer acquisition times or a higher LOD.

### 2.3. Repeatability

The repeatability of the quantitation of the minor enantiomer depends on the relative concentration of that enantiomer. Therefore, the repeatability was tested at two different concentrations. Both the solution containing 23.7% of the S enantiomer (by experiment) and the solution containing 6.9% of the S enantiomer (by experiment) were tested 6 times. For this test, a larger volume of CDCl_3_ solution containing the desired amounts of S and R enantiomer was prepared, and for each replicate 0.6 mL of the same solution was pipetted to an amount of approximately 40 mg of TFAE. In this way, the preparation of artificial mixtures is the same for all replicates, while the precise amount of TFAE, the preparation of the NMR solution and the recording and evaluation of the NMR spectra are the variables tested in the repeatability experiment. Table 2 shows the individual results per replicate, the average and standard deviation. A relative standard deviation of 0.3% and 1.4% for the minor enantiomer were found for the two abovementioned concentrations, which represents an excellent result for the repeatability.

## 3. Discussion

^1^H NMR has been the preferred NMR technique for enantiodiscrimination, and ^13^C NMR has been neglected for a long time. This is probably due to the fact that ^13^C NMR is less sensitive than ^1^H NMR, and also due to the general idea that ^13^C NMR cannot be applied for quantitative purposes. Nowadays, sensitivity of ^13^C NMR on high-field instruments equipped with a cryoprobe is more than sufficient, and the data presented above show convincingly that quantitation is very accurate and precise when stereoisomers of the same compound are compared. If a smaller amount of the chiral compound is available, and the sensitivity of ^13^C NMR is a limitation, methods based on Dynamic Nuclear Polarization (DNP) can overcome this limitation [35]. However, the instrumentation is expensive and will not be available in standard laboratories in the near future.

There are several situations where ^1^H NMR fails to show good separation of the enantiomeric signals, for instance in the case of complex mixtures of multiple stereoisomers of compounds with more than one chiral center, but also when the compound of interest lacks protons, or when a proton close to the chiral center does not show the desired splitting. One should keep in mind that the splitting of signals is caused by the strong shielding and deshielding effects of the anthryl moiety of TFAE (or of an aromatic ring from a different CSA). The magnitude of the shielding or deshielding effect depends on the actual geometry of the association complex formed, and it is difficult and time-consuming to predict this effect with high accuracy. In special situations, the proton of the compound of interest might be in a region where the shielding or deshielding effect is zero. Furthermore, the actual splitting of the signal depends on the difference of the shielding/deshielding effect on the R and on the S compound. By coincidence, the magnitude of the effect can be the same for both compounds.

Apparently, in the present case, by coincidence, no signal separation is observed on the C2 proton, which is rather unexpected, because C2 is the chiral center of the molecule. In addition, the splitting of the C2 carbon is only very small. The chance to find a suitable probe that resides in a favorable region with respect to the aromatic rings is higher in ^13^C NMR than in ^1^H NMR, and, at the same time, another big advantage is that a very high resolution is obtained. This is best illustrated in Figure 4.

## 4. Materials and Methods

R/S-mandelonitrile and R-mandelonitrile were purchased from Alfa Aesar (Haverhill, MA, USA), L08698 and H56658, respectively. (S)-(+)-1-(9-anthryl)-2,2,2-trifluoroethanol (TFAE) was purchased from Sigma-Aldrich (St. Louis, MO, USA). Samples were dissolved in CDCl_3_ (Cambridge Isotope Laboratories, Tewksbury, MA, USA).

Approximately 4 mg of mandelonitrile and 40 mg of TFAE were dissolved in 0.6 mL of solvent. These concentrations correspond to molar concentrations of 50 mM and 240 mM, respectively. Thus, the molar excess of TFAE was 5×.

Preparation of artificial mixtures and other critical weighing was done using a Mettler Toledo XP205 balance (Columbus, OH, USA) with a repeatability of 0.015 mg.

NMR spectra were recorded on a Bruker Avance III 700 MHz NMR spectrometer (Billerica, MA, USA), equipped with a cryogenically cooled 5 mm TCI probe. The low-temperature version of this probe was used, which can be operated in a temperature range of 240–340 K. Standard conditions for NMR data acquisition were as follows: spectra were recorded with 256 scans and a delay of 3 s between pulses in order to avoid heating of the sample, resulting in an acquisition time of 20 min. A time domain of 64 K data points was used, and free induction decays (FIDs) were zero filled to 128 K data points. The sweep width was reduced to 100 ppm, covering the range from 150 ppm–50 ppm. In this way, a high digital resolution was obtained of 0.13 Hz/pt. A sample temperature of 260 K was chosen as the best temperature for separation of signals, and all spectra used for the validation were recorded at this temperature. All spectra were obtained with power-gated decoupling using the standard Bruker (Billerica, MA, USA) pulse program zgpg.

T1 values were obtained by the standard Bruker program t1irpg. All data were processed with Bruker Topspin 3.5.

Before Fourier Transformation, a Gaussian filter was applied with parameters LB = −0.3, GB = 0.25.

Chemical shifts were referenced relative to tetramethylsilane (TMS). The center peak of CDCl_3_ was assigned the value 77.04 ppm (relative to TMS) once, and, in all other spectra, this peak was used for referencing.

## 5. Conclusions

The data show that ^13^C NMR is very quantitative when applied for the determination of enantiomeric ratios. Even though ^13^C NMR is less sensitive than ^1^H NMR, on modern NMR instruments, the sensitivity is more than sufficient, and advantages are: very high resolution, and standard broadband decoupled spectra, hence better separation of signals and precise integration, and a higher chance to find a suitable probe in the molecule to determine the enantiomeric ratio. In many cases, these advantages are more important than the disadvantage of a lower sensitivity.

## Figures and Tables

**Table 1:** Percentage of S enantiomer in artificial mixtures. A: by weight of the two ingredients. B: by weight after correction for the enantiomeric purity and the chemical purity (predicted). C: by ^13^C NMR experiment at 260 K.

A	B	C
% S by Weight	%S Predicted (after Correction)	% S by Experiment
0	2.4	2.4
4.3	6.8	6.9
10.5	13.1	13.0
21.7	24.1	23.7
40.8	41.9	41.8
50	50.1	50.1

**Table 2:** Repeatability of the quantitation of the minor enantiomer at two different concentrations. NMR spectra recorded at 260 K.

Replicate nr	S Enantiomer (%)	S Enantiomer (%)
1	23.7	6.9
2	23.8	6.8
3	23.8	6.7
4	23.9	6.7
5	23.9	6.7
6	23.8	6.8
		
Average	23.8	6.8
Standard deviation	0.1	0.1
Relative standard deviation (%)	0.3	1.4
